# The impact of utilizing inclusive leadership among nurses during crises: A multisite comparative study

**DOI:** 10.25122/jml-2023-0159

**Published:** 2023-09

**Authors:** Hasan Abualruz, Suhair Hussni Al-Ghabeesh, Heba El-Gazar, Nazih Abu Tabar, Hussain Al-Sharyah, Rozan Al-Sarayreh, Ali Abousoliman

**Affiliations:** 1 Faculty of Nursing, Al-Zaytoonah University of Jordan, Amman, Jordan; 2 Faculty of Nursing, Port Said University, Port Fuad, Egypt; 3 Fatima College of Health Sciences, Al Ain, United Arab Emirates; 4 AL-Ghad International Colleges for Applied Medical Sciences, Najran, Saudi Arabia; 5 Faculty of Nursing, Al-Zaytoonah University of Jordan, Amman, Jordan; 6 Prince Sattam Bin Abdulaziz University, Riyadh, Saudi Arabia; 7 Faculty of Nursing, Kafr Elsheikh University, Kafr El Sheikh, Egypt

**Keywords:** inclusive leadership, job satisfaction, psychological distress

## Abstract

Nurses' psychological wellness and satisfaction are threatened by exposure to many stressors. Adopting a promising leadership style has beneficial impacts at different levels, especially during crises. This study aimed to examine the impact of inclusive leadership on nurses' satisfaction and psychological distress during crises, focusing on three Arabic countries. A cross-sectional descriptive design was utilized to meet the study goal. Data were collected electronically in the three countries using the Kessler Psychological Distress Scale (K-10), Minnesota Satisfaction Questionnaire, and Carmel's Inclusive Leadership Scale. Two hundred seventy-four participants fully responded: 90 from Egypt, 82 from Saudi Arabia, and 102 from Jordan. Descriptive statistics, Pearson R, Spearman Rho, Point biserial, and ANOVA tests were used to answer the research questions. There were statistically significant differences between countries in the mean scores of inclusive leadership and psychological distress. In addition, statistically significant relationships between inclusive leadership, psychological distress, and job satisfaction were found. The study focused on the importance of approaching inclusive leadership to increase employee satisfaction, reduce psychological distress, and achieve organizational goals.

## INTRODUCTION

Nursing is widely recognized as one of the most demanding professions in the world, as nurses are exposed to physical and psychological problems due to the nature of their work [1]. Nurses, in particular, have faced heightened risks during the Coronavirus Disease (COVID-19) pandemic, with increased exposure to life-threatening infections. This exposure negatively impacts the well-being of nurses, largely because highly contagious pathogens pose a significant threat. Moreover, nurses often work in isolation units or other settings where direct contact with infected individuals is common [2]. Psychological distress is widespread among nurses as researchers have linked depression and anxiety to exposure to life-threatening conditions and other serious work-related stressors [3].

Leaders’ roles can positively influence the level of psychological wellness among nurses. Fair leadership has been shown to minimize the symptoms of depression and anxiety in contaminated work environments [4]. Leaders and organizations need to spot factors and adopt leadership styles that promote the psychological wellness of nurses in such complicated environments since they affect the quality of care provided and the main organizational outcomes. One of the modern influencing leadership styles in such situations is Inclusive Leadership [5].

Inclusive leadership helps alleviate mental pressure among employees [6]. Leaders adopting inclusiveness add value to the organization by treating employees from diverse cultural backgrounds effectively, implementing a culture of equity and fairness, and initiating a healthy work environment that promotes psychological wellness [7].

It is undisputed that workplace job satisfaction is important to retain and develop human capital in the business strategy [8]. Job satisfaction in nursing is related to role performance, provided care quality, and the satisfaction of clients [9]. However, nurses are often unsatisfied due to many internal and external factors [10]. Inclusive leadership integrates many concepts that play dynamic roles in incorporating nurses in decisions and mapping their career plans, thus promoting job satisfaction among nurses. The COVID-19 pandemic shed light on this issue and its impacts on nurses and organizations [11].

Effective leadership inspires a constructive work environment that ensures the optimal achievements of each person [12]. Thus, adopting a promising leadership style such as inclusive leadership is necessary to improve nurses' job satisfaction, psychological wellness, and ability to cope with stressors, achieve organizational goals, and improve the quality of care [13]. The study examined the impact of inclusive leadership on nurses' satisfaction and psychological distress during the COVID-19 pandemic in three major Arabic countries: Egypt, Saudi Arabia, and Jordan.

This study made its significance by shedding light on the importance of implementing inclusive leadership in hospitals in the Arabic region, whereas no such studies have been conducted. Furthermore, it is the first study applied at the regional level (3 main Arabic countries), in which the results were compared and justified. In addition, inclusive leadership impact was studied on two of the most important outcomes affecting nursing retention, quality of provided care, and patient safety. Moreover, the study was conducted during the COVID pandemic, when the surrounding conditions and employees' demands were different and increased.

## MATERIAL AND METHODS

### Design, setting, and sample

A cross-sectional-descriptive design was utilized to examine the impact of inclusive leadership on psychological distress and job satisfaction among nurses during the COVID-19 pandemic and compare the results between Egypt, Saudi Arabia, and Jordan. The authors developed an online questionnaire and distributed it to the most popular nursing groups in the three countries through various social media platforms, including Facebook, Twitter, and WhatsApp.

The G*Power 3 software [14] was used to compute the sample size. Based on G power calculations, a total of 200 participants was required minimum for an α of .05, a power of 0.80, and a medium effect size of 0.25. The link to the questionnaire was shared with the most popular nursing groups on social media platforms in the three countries. A total of 274 participants fully responded and answered all questions in the questionnaire, with 90 participants from Egypt, 82 from Saudi Arabia, and 102 from Jordan. All participants met the following inclusion criteria: working directly with patients during the COVID-19 pandemic, being able to read and write both Arabic and English and holding at least a diploma degree in nursing. Nurses not assigned to care for COVID-19 patients were excluded from the study.

### Data collection

The data collection process started at the beginning of November, 2021, and lasted one month. Data were collected electronically by sharing the questionnaire link to the most popular nursing groups on social media platforms (Facebook, Twitter, and WhatsApp) across the three countries: Egypt, Saudi Arabia, and Jordan. Each author shared the questionnaire's link to the most popular nursing groups in his country. Interested individuals who received the invitation could access the questionnaire, review instructions and study details, and provide electronic consent before responding to the survey. The questionnaire was created using Survey Sparrow, an online platform that facilitates the creation of electronic surveys, ensuring ease of use, accessibility, and sharing.

### Operational definitions

*Inclusive leadership*: Inclusive leadership was assessed based on participants' responses to the 9 items in Carmel's Inclusive Leadership Scale. The scoring system ranges from 1 to 63, each item rated on a 7-point Likert scale.

*Psychological distress*: Psychological distress was evaluated through participants' responses to the 10 items in the Kessler Psychological Distress Scale (K-10). Each item is rated on a 5-point Likert scale. The total score for psychological distress is calculated out of 50.

*Job satisfaction*: Job satisfaction was determined based on the participant's responses to the Minnesota Satisfaction Questionnaire. Each item in this questionnaire is rated on a 5-point Likert scale. The overall job satisfaction score is calculated out of 100.

### Data collection tools

The authors developed a demographic questionnaire only for this study, covering data on age, gender, years of experience, educational level, country, job designation, and working hours per week. To assess psychological distress (depression and anxiety), the authors used the Kessler Psychological Distress Scale (K-10), a valid and reliable 10-item-5 points Likert scale. Scores below 25 indicate normal or mild symptoms, 25-29 suggest moderate, and 30-50 indicate severe symptoms. K-10 is a valid and reliable tool; the reported Cronbach's alpha is 0.88 [15]. In this study, the K-10 showed good consistency (Cronbach's alpha: 0.82).

Job satisfaction was measured using the Minnesota Satisfaction Questionnaire (MSQ), a valid and reliable 20-item-5-point Likert scale. Scores range from 20 to 100, with higher scores indicating greater satisfaction. Reported Cronbach's alpha ranged from 0.85-0.91 [16]. In this study, the Cronbach’s alpha was 0.85. Inclusive leadership was assessed using Carmel's Inclusive Leadership Scale, a valid and reliable 9-item-7 points scale with a reported Cronbach's alpha of 0.94. Scores range from 7 to 63 [17]. In this study, the Cronbach’s alpha was 0.88.

### Analysis and research questions

To meet the purpose of the study, the researchers addressed the following research questions:

1. What is the extent of inclusive leadership implementation in clinical settings in Egypt, Saudi Arabia, and Jordan during the COVID-19 pandemic?
2. What are the levels of psychological distress and job satisfaction among nurses in Egypt, Saudi Arabia, and Jordan during the COVID-19 pandemic?
3. Are there relationships between the socio-demographic variables and inclusive leadership, psychological distress, and job satisfaction among nurses in Egypt, Saudi Arabia, and Jordan during the COVID pandemic?
4. Are there relationships between inclusive leadership, psychological distress, and satisfaction among nurses in Egypt, Saudi Arabia, and Jordan during the COVID pandemic?
5. Are there differences in the means of inclusive leadership, psychological distress, and job satisfaction among nurses in Egypt, Saudi Arabia, and Jordan during the COVID pandemic?

All collected data were complete, with no missing entries, as the survey website accepted only fully completed questionnaires. Data was transferred to the Statistical Package for the Social Sciences (SPSS) version 23. The characteristics of participants (age, gender, years of experience, educational level, country, job designation, and working hours per week) were described using mean, median, standard deviation, range, frequency, and percentages. The psychological distress, inclusive leadership, and job satisfaction levels (questions one and two) were assessed using mean, median, standard deviation (SD), and range. The relationship between variables (questions number three and four) was investigated using the Pearson correlation coefficient, Spearman's rank-order correlation coefficient, and Point Biserial correlations. Differences in means for inclusive leadership, job satisfaction, and psychological distress among Egypt, Saudi Arabia, and Jordan (question number five) were examined using analysis of variance (ANOVA).

## RESULTS

### Sample characteristics

A total of 274 participants responded to the electronic questionnaire: 90 participants from Egypt, 82 from Saudi Arabia, and 102 from Jordan. The mean age of participants was 33 (±5.3) years, and the mean experience years was 9.7 (±5.6). Most respondents were male, 173 (63.1%), and 101 (36.9%) were female. A total of 192 (70.1%) participants hold a bachelor's degree, 74 participants (27%) hold post-graduate degrees, and only 8 (2.9) participants hold diplomas. Of the participants, 197 (71.9%) worked as bedside nurses or senior nurses, and 77 (28.1%) worked on supervisory levels. The total working hours for participants were as follows: 71 (25.9%) worked 40 hours or less, and the remaining 203 participants (74.1%) worked more than 40 hours. Table 1 describes the details of sample characteristics.

**Table 1:** Sample characteristics (N=274)

Variable	Egypt (N=90)				Saudi Arabia (N=82)				Jordan (N=102)			
**Age** All countries mean (SD)=33.0(5.3)	Mean (SD)	Median		Range	Mean (SD)	Median		Range	Mean (SD)	Median		Range
	32.3 (5.2)	33		23-50	33.4 (4.7)	33		26-50	33.2 (5.8)	33		22-50
**Experience** All countries mean (SD)=9.7(5.6)	Mean(SD)	Median		Range	Mean(SD)	Median		Range	Mean(SD)	Median		Range
	9.2 (5.6)	9		1-30	10.4 (5.1)	10		2-30	9.6 (6)	10		1-30
**Gender**	Male Frq (%)		Female Frq (%)		Male Frq (%)		Female Frq (%)		Male Frq (%)		Female Frq (%)	
	60 (66.7%)		30 (33.3%)		51 (62.2%)		31 (37.8%)		62 (60.8%)		40 (39.2%)	
**Job Designation**	Bedside Frq (%)		Supervisory levelFrq (%)		Bedside Frq (%)		Supervisory levelFrq (%)		Bedside Frq (%)		Supervisory levelFrq (%)	
	55 (61.1%)		35 (38.9%)		64 (78%)		18 (22%)		78 (76.5%)		24 (23.5%)	
**Working hours**	<40 hours		≥40 hours		<40 hours		≥40 hours		<40 hours		≥40 hours	
	24 (26.7%)		66 (73.3%)		16 (195%)		66 (80.5%)		31 (30.4%)		71 (69.6%)	
**Education level**	Under-gradute		Post- graduate		Under-gradute		Post- graduate		Under-gradute		Post- graduate	
	60 (66.7%)		30 (33.3%)		60 (73.2%)		22 (26.8%)		80 (78.4%)		22 (21.6%)	

### Inclusive leadership, psychological distress, and job satisfaction

The mean inclusive leadership score among all countries was 44.1 (±11.8), whereas the highest possible score is 63. The mean job satisfaction score was 63.0 (±15.0), whereas the highest possible score was 100. The psychological distress mean was 27.1 (±8.3), whereas 50 is the worst psychological distress score. The majority of participants, 258 (94.2%), had psychological distress ranging from moderate to severe. Details about each country's statistics are presented in Table 2.

**Table 2:** Inclusive Leadership, psychological distress, and job satisfaction scores (N=274)

Variable	Mean (SD)	Median	Range	Frequency %
Inclusive leadership (All countries)	44.1 (11.8)	46	14-63	
Egypt	48.0 (11.3)	49	14-63	
Saudi Arabia	40.3 (11.5)	42	15-63	
Jordan	43.8 (11.6)	46	14-63	
Job satisfaction ( All countries)	63.0 (15.0)	63	22-100	
Egypt;	65.3 (16.2)	66	22-100	
Saudi Arabia	62.0 (13.0)	62	33-90	
Jordan	61.8 (15.3)	61	27-92	
Psychological distress ( All countries)	27.1 (8.3)	27	10-50	
- normal or mild				16 (5.8%)
- moderate				144 (52.6%)
- high				114 (41.6%)
Egypt (n=90)	26.0 (9.1)	24	10-50	
- normal or mild				8 (8.8%)
- moderate				50 (55.6%)
- high				32 (35.6%)
Saudi Arabia (n=82)				
- normal or mild	29.0 (8.0)	30	14-42	1 (1.2%)
- moderate				39 (47.6%)
- high				42 (51.2%)
Jordan (n=102)				
- normal or mild	26.5 (7.7)	26	11-42	7 (6.9%)
- moderate				55 (53.9%)
- high				40 (39.2%)

### Relationships between the study variables

Statistically significant inverse relationships between psychological distress and the following demographic variables: age (r=-0.17), experience (r=-0.14), education (r=-0.18), and job designation (r=-0.13) were found. Those with postgraduate degrees and working at supervisory levels had significantly less psychological distress. Job satisfaction had statistically significant positive relationships with job designation (r=0.12) and working hours (r=0.19). Participants working less than 40 hours per day and those working in supervisory positions had higher job satisfaction scores. Inclusive leadership was negatively correlated with age, years of experience, level of education, and gender (r=-0.14, -0.16, -0.16, -0.25) respectively. Female participants and participants with post-graduate degrees responded more positively to the inclusive leadership scale. Details are presented in Table 3.

**Table 3:** Relationshipsabetween study variables

Variable	Inclusive leadership	Psychological distress	Job satisfaction
**Inclusive leadership**	-	-0.28^**^	0.52^**^
**Psychological distress**	-0.28^**^	-	-0.18^**^
**Job satisfaction**	0.52^**^	-0.18^**^	-
**Age**	-0.14^*^	-0.17^**^	-
**Experience**	-0.16^*^	-0.14^*^	-
**Gender**	-0.25^**^	-	-
**Job designation**	-	-	0.12^*^
**Working hours**	-	-0.13^*^	0.19^**^
**Education**	-0.16^**^	-0.18^**^	-

* : Significant at alpha 0.05; ^**^: Significant at alpha <0.001

A statistically significant negative relationship between inclusive leadership and psychological distress was indicated in the results (r=-0.28), and a statistically significant positive relationship between inclusive leadership and job satisfaction was found (r=0.52) as well. Furthermore, a statistically significant negative relationship was found between job satisfaction and psychological distress (r=-0.18). Details are presented in Table 3.

### Comparisons between countries

Before conducting the main analysis, there were no significant differences in demographics among countries (all p values >0.05). When comparing the means of inclusive leadership and psychological distress among countries, statistically significant differences were observed in group means (F=9.72, p<0.001), (F=3.37, p=0.04) respectively. Post-hoc Scheffe tests revealed that inclusive leadership scores were highest in Egypt, followed by Jordan and Saudi Arabia. In contrast, in Saudi Arabia, psychological distress scores were highest, followed by Jordan and Egypt.

On the other hand, there was no statistically significant difference in job satisfaction means among countries (F=1.62, p=0.20). Although there was no significant difference, the Scheffe test results indicated that nurses in Egypt reported the highest job satisfaction scores, followed by those in Saudi Arabia and Jordan. Details are presented in Table 4.

**Table 4:** Comparisons across groups

Variable	df	F	p-value
**Inclusive leadership**	2	9.72	^**^<0.001
**Pscyhological distress**	2	3.37	^*^0.04
**Job satisfaction**	2	1.62	0.20

* : Significant at alpha 0.05; ^**^: Significant at alpha <0.001

## DISCUSSION

The present study aimed to assess the influence of inclusive leadership on nurses' job satisfaction and psychological distress during the COVID-19 pandemic in three major Arabic countries: Egypt, Saudi Arabia, and Jordan. Previous studies focused on different leadership styles and their impacts under normal circumstances, and little is known about the roles of leadership styles during pandemics and crises [18]. This study worked on filling this gap by investigating the impact of inclusive leadership during significant workplace changes induced by the COVID-19 pandemic. Furthermore, this is a multi-site study that collected data from three Arabic countries to confirm the results, being the first study conducted at the regional level within the Arab nations.

The results of the study showed that the mean inclusive leadership score among the three countries was around the midpoint, indicating that nurses perceived the behaviors of their leaders as moderately inclusive during the pandemic. These results are relatively lower than those reported in South Korea [17] and Wuhan, China [19]. No data were available about the situation in Arabic countries for comparison. This acceptable moderate level of perceived inclusive leadership could be attributed to the unique circumstances created by the COVID-19 pandemic. Crises of this magnitude may prompt nursing leaders to adopt more open, accessible, and available communication approaches with their nursing staff, ultimately leading to an increase in perceived inclusive leadership qualities [18, 20].

The results showed that younger females with less experience and lower education tended to perceive their nursing leaders as more inclusive. This result agrees with another study, which also observed an increase in perceptions of inclusiveness among younger and less experienced female nurses, although it did not find a significant association with education level [21]. The negative correlation between inclusive leadership and education might be justified by the advanced abilities of nurses with postgraduate degrees to critique and appraise the organizations and their advanced and integrated vision toward leadership practices.

Egyptian nurses reported a higher level of inclusive leadership than those from Jordan and Saudi Arabia. Differences in workplace conditions in terms of organizational structure and accreditation bodies of control and the nature of humans in perceiving stressors in difficult situations may explain the variation. Egyptian nurses have solid cultural relationships with colleagues and leaders, which makes them accessible and available all the time [22]. On the other hand, the lower level of perceived inclusive leadership in Saudi Arabia compared to other countries might be linked to the severe shortage of Saudi nurses, leading the country to rely on nurses from various nations and cultural backgrounds.

The mean score of job satisfaction among the three countries included in the study was slightly above its midpoint, suggesting a moderate level of nurses' job satisfaction. These results are congruent with those reported in Iran [23] and Iraq [24], but higher than those reported in Beijing [25], the Philippines [26], and the United Kingdom [27]. Since nurses are considered frontline healthcare professionals, efforts are still needed to increase nurses' job satisfaction [28, 29].

In the same context, the study found that nurses working less than 40 hours per day and nurses working in supervisory positions had higher job satisfaction scores. This could be explained by the fewer working hours and the nature of the supervisory positions in which less physical effort is needed. Previous studies showed contradictory results, where job satisfaction significantly differed based on nurses' sex, age, and years of experience, but not necessarily according to job designation or working hours [30]. This contradiction is accepted since the cultural backgrounds are different between the studies.

Specifically, Egyptian nurses reported a higher level of job satisfaction compared to nurses from Saudi Arabia and Jordan. This could be due to the reliance of Egypt on local nurses living with their families, in contrast to nurses in other countries who often work as expatriates. Furthermore, the higher inclusive leadership behaviors in Egypt than in other countries is an additional justification, as job satisfaction was positively correlated with inclusive leadership.

The results showed that the majority of nurses in the three countries suffer from moderate to severe levels of psychological distress. These results align with results reported in Malaysia [31] and China [28]. This underscores the need for nursing administrators to develop a well-structured workplace protocol to alleviate psychological distress among nurses. This protocol should contain guidelines to curb psychological distress levels, measures to identify suitable nurses, and actions to handle affected nurses. Adopting inclusive leadership is an important, affordable option, as this study showed a significant negative relationship between psychological distress and inclusive leadership.

Additionally, it was found that older nurses, expert nurses, nurses with higher education, and nurses in supervisory positions had significantly less psychological distress. This finding aligns with existing literature, as it is widely recognized that nurses with greater age, experience, education, and supervisory responsibilities tend to have greater expertise in coping with workplace stressors, which are reflected in their psychological wellness [28].

Specifically, Saudi Arabian nurses reported higher psychological distress than those from Jordan and Egypt. This finding might be due to the extreme cultural diversity in the workplace in Saudi Arabia. Recent literature showed that Saudi nurses comprise around 38% of the total nurses in the kingdom, and the other proportion considered foreign nurses from countries such as the Philippines, India, and Malaysia [32]. Another contributing factor may be the challenges discussed in another study [33], including a lack of specialty, education, and research opportunities, which could contribute to increased psychological distress among Saudi nurses.

### Implications for nursing management

Nurses are the frontline healthcare professionals who work against the COVID-19 pandemic. The findings of this study offer valuable insights for nursing and hospital leaders in the Arabic region and globally, emphasizing the significance of adopting inclusive leadership. This leadership style can positively impact nurses' job satisfaction and psychological wellness, specifically after the COVID-19 battle. This recommendation is supported by the results of another study [19], which found that inclusive leadership could curb psychological distress caused by the COVID-19 pandemic and foster a culture of psychological safety among nurses after the COVID-19 crisis. Improving nurses' psychological wellness and satisfaction will be very helpful in meeting the organizational, national, and regional contemporary health vision by improving the quality of the care provided and patients' safety [34, 35].

## CONCLUSION

Crises management is an integral part of organizational management, and in today's globalized world, diversity has become a prevalent feature within organizations. Managing crises in culturally diverse organizations is a major challenge facing leaders during those times. Leaders need to recognize how to execute their roles effectively to gain the optimal advantages of diversity, treat employees effectively, overcome crises, and achieve the intended organizational goals. Practicing inclusive behaviors by leaders is a recommended modern model to achieve such goals. Adopting inclusive leadership will be reflected positively in nurses' satisfaction, psychological wellness, organizational integrity, overcoming crises, and thus meeting the health vision, especially during crises.

## Data Availability

The datasets used and/or analyzed during the current study are available from the corresponding author upon reasonable request.

## Personal thanks

We would like to thank the study participants for their time.
